# Support for Transition from Adolescent to Adult Health Care Among Adolescents With and Without Mental, Behavioral, and Developmental Disorders — United States, 2016–2017

**DOI:** 10.15585/mmwr.mm6934a2

**Published:** 2020-08-28

**Authors:** Rebecca T. Leeb, Melissa L. Danielson, Rebecca H. Bitsko, Robyn A. Cree, Shana Godfred-Cato, Michelle M. Hughes, Patrick Powell, Bradley Firchow, Laura C. Hart, Lydie A. Lebrun-Harris

**Affiliations:** ^1^Division of Human Development and Disability, National Center on Birth Defects and Developmental Disabilities, CDC; ^2^Division of Birth Defects and Infant Disorders, National Center on Birth Defects and Developmental Disabilities, CDC; ^3^Oglethorpe University, Atlanta, Georgia; ^4^The Ohio State University College of Medicine, Nationwide Children’s Hospital, Columbus, Ohio; ^5^Maternal and Child Health Bureau, Health Resources and Services Administration, Washington, DC.

Clinical guidelines recommend that primary care providers (PCPs) provide guidance and support to ensure a planned transition from pediatric to adult health care for adolescents, beginning at age 12 years (*1*). However, most adolescents do not receive the recommended health care transition planning (*2*). This is particularly concerning for adolescents with diagnosed mental, behavioral, and developmental disorders (MBDDs) (*3*), who account for approximately 20% of U.S. adolescents (*4*). Childhood MBDDs are linked to increased long-term morbidity and mortality; timely health care transition planning might mitigate adverse outcomes (*5*,*6*). CDC analyzed pooled, parent-reported data from the 2016 and 2017 National Survey of Children’s Health (NSCH), comparing adolescents, aged 12–17 years, with and without MBDDs on a composite measure and specific indicators of recommended health care transition planning by PCPs. Overall, approximately 15% of adolescents received recommended health care transition planning: 15.8% (95% confidence interval [CI] = 14.1%–17.5%) of adolescents with MBDDs, compared with 14.2% (95% CI = 13.2%–15.3%) of adolescents without MBDDs. Relative to peers without MBDDs and after adjusting for age, adolescents with anxiety were 36% more likely to receive recommended health care transition planning, and those with depression were 69% more likely; adolescents with autism spectrum disorder (ASD) were 35% less likely to receive such transition planning, and those with developmental delay* were 25% less likely. Fewer than 20% of adolescents with MBDDs receiving current treatment met the transition measure. These findings suggest that a minority of adolescents with MBDDs receive recommended transition planning, indicating a potential missed public health opportunity to prevent morbidity and mortality in a population at high risk for health care disengagement (*1*). Improving access to comprehensive and coordinated programs and services,^†^ as well as increasing provider training concerning adolescents’ unique mental and physical health care needs (*7*), could help increase the number of adolescents benefiting from successful health care transitions (*4*).

NSCH, a nationally representative, cross-sectional survey of parents and guardians, is funded and directed by the Health Resources and Services Administration’s Maternal and Child Health Bureau (HRSA MCHB) and conducted by the U.S. Census Bureau.^§^ MBDDs were identified based on parents’ affirmative responses to the question “Has a doctor or other health care provider ever told you that this child has (specified disorder)?” and whether the child currently had the MBDD; adolescents with no reported MBDDs constituted the comparison group. MBDDs were categorized as “behavioral disorders” (attention-deficit/hyperactivity disorder [ADHD], behavioral or conduct problems, or Tourette syndrome), “emotional disorders” (anxiety problems or depression), and “developmental disorders” (ASD, learning disability, intellectual disability, developmental delay, or speech or other language disorder).^¶^ Parents reported whether each current MBDD was “mild,” “moderate,” or “severe.” Treatment was based on whether 1) the child had taken any medication for emotional, concentration, or behavioral difficulties in the past 12 months or was currently taking medication for ADHD or ASD; or 2) the child was currently receiving behavioral services, such as speech, occupational, or behavioral therapy; treatment or counseling from a mental health professional; or behavioral treatment for ADHD or ASD in the past 12 months.

Consistent with previous research (*2*,*3*), a three-element** transition measure aligning with the HRSA MCHB National Performance Measure^††^ for health care transition planning was used: 1) any time alone with PCP at last preventive visit^§§^; 2) PCP worked with the adolescent to gain health management skills or understand health care changes occurring at age 18 years; and 3) PCP discussed the shift to an adult PCP. Adolescents who met all three elements met the transition measure.

Weighted response rates overall for NSCH were 40.7% for 2016 and 37.4% for 2017.^¶¶^ Analyses included 29,286 adolescents aged 12–17 years with data*** for current MBDDs and transition questions. Weighted prevalence estimates for adolescents meeting the transition measure were compared by MBDD status across sociodemographic subgroups using unadjusted prevalence ratios (PRs) and 95% Clopper-Pearson CIs. Prevalences of meeting the transition measure and the three elements were calculated by MBDD status and MBDD category and compared using age-adjusted prevalence ratios (aPRs)^†††^ and 95% CIs. Analyses were conducted using SAS-callable SUDAAN (version 11.0.3; RTI International) to accommodate the complex sample design and sampling weights.

Overall, 14.6% (95% CI = 13.7%–15.5%) of adolescents met the transition planning measure; 24.2% (95% CI = 23.1%–25.4%) had one or more MBDDs. Meeting the transition measure did not differ significantly by MBDD status (with MBDDs = 15.8%, without MBDDs = 14.2%) (Table 1), but subgroup differences were detected. Adolescents with MBDDs were more likely to meet the transition measure than were those without MBDDs among females (19.0% versus 13.6%), non-Hispanic whites (17.3% versus 14.8%), other non-Hispanic race/ethnicity groups (20.2% versus 13.4%), those with private insurance (17.3% versus 14.4%), and those without insurance (14.8% versus 6.8%). Older adolescents (aged 15–17 years) were more likely than were younger adolescents to meet the transition measure regardless of MBDD status (Supplementary Figure 1, https://stacks.cdc.gov/view/cdc/92137). Adolescents with emotional disorders tended to be older than were adolescents with behavioral or developmental disorders (Supplementary Figure 2, https://stacks.cdc.gov/view/cdc/92137). Adolescents with MBDDs were more likely to have time alone with their PCP at their last preventive visit (aPR = 1.18; 95% CI = 1.10–1.27) and work with their PCP to gain health management skills or understand health care changes occurring at age 18 years (aPR = 1.08; 95% CI = 1.02– 1.13) than were adolescents without MBDDs; however, they were less likely to have discussed the shift to an adult provider with their PCP (PR = 0.90; 95% CI = 0.85–0.96) (Table 2).

**TABLE 1:** Percentage of U.S. adolescents aged 12—17 years meeting the transition planning measure, by mental, behavioral, or developmental disorder (MBDD) status among sociodemographic subgroups — National Survey of Children’s Health, United States, 2016–2017

Characteristic	With MBDD		No MBDD		Comparison of adolescents with and without MBDDs*
	No. (unweighted)	% (95% CI)	No. (unweighted)	% (95% CI)	PR (PR 95% CI)
**Total**	7,622	15.8 (14.1–17.5)	21,664	14.2 (13.2–15.3)	1.11 (0.98–1.26)
**Sex**					
Male	4,187	13.3 (11.2–15.7)	10,714	14.8 (13.2–16.4)	0.90 (0.74–1.10)
Female	3,435	19.0 (16.4–21.8)	10,950	13.6 (12.3–15.0)	1.40** (1.18–1.66)
**Age group (yrs)**					
12–14	3,345	10.2 (8.0–12.6)	9,725	8.8 (7.7–10.0)	1.16 (0.90–1.49)
15–17	4,277	21.5 (19.0–24.1)	11,939	19.5 (17.8–21.3)	1.10 (0.95–1.27)
**Race/Ethnicity**					
White, non-Hispanic	5,662	17.3 (15.3–19.6)	15,212	14.8 (13.8–15.7)	1.18** (1.02–1.35)
Black, non-Hispanic	467	16.5 (11.6–22.6)	1,342	14.1 (11.1–17.6)	1.18 (0.80–1.73)
Hispanic	750	9.0 (6.0–12.9)	2,310	13.5 (10.6–16.8)	0.67 (0.44–1.03)
Other, non-Hispanic	743	20.2 (15.3–25.9)	2,800	13.4 (11.2–15.8)	1.51 (1.11–2.05)
**Urbanicity^†^**					
Living outside an MSA (rural)	1,500^§^	19.5 (15.9–23.6)	4,000^§^	15.8 (13.9–18.0)	1.23 (0.97–1.56)
Living in an MSA (urban or suburban)	6,100^§^	15.2 (13.4–17.1)	17,500^§^	14.0 (12.9–15.2)	1.09 (0.94–1.26)
**Health insurance status**					
Public insurance only	1,851	13.7 (11.0–16.8)	2,938	15.3 (12.6–18.4)	0.90 (0.68–1.18)
Private insurance only	4,884	17.3 (15.0–19.8)	16,856	14.4 (13.4–15.5)	1.20** (1.03–1.40)
Public and private insurance	507	12.0 (8.2–16.6)	550	14.0 (8.5–21.2)	0.85 (0.50–1.47)
Unspecified insurance	64	32.7 (13.1–58.1)	210	20.2^¶^ (4.7–47.6)	1.61 (0.49–5.31)
No insurance	262	14.8 (8.0–24.2)	941	6.8 (4.6–9.6)	2.18** (1.17–4.07)

**Abbreviations:** CI = confidence interval; MSA = metropolitan statistical area; PR = prevalence ratio.

* Reference group is adolescents without MBDDs.

^†^ Residence in or not in an MSA was used as a proxy for urbanicity. Adolescents living in an MSA were considered to be living in an urban or suburban area, and adolescents not living in an MSA were considered to be living in a rural area. The U.S. Census Bureau reviewed the urban/rural status estimates for unauthorized disclosure of confidential information and approved the disclosure avoidance practices applied to the data release (Approval ID CBDRB-FY20-POP001–0053).

^§^ The unweighted n for each urbanicity subgroup has been rounded to the nearest hundred to follow U.S. Census Bureau disclosure avoidance practices for data release.

^¶^ Estimate does not meet the National Center for Health Statistics standards of precision and should be interpreted with caution. The absolute width of the 95% CI is >30 percentage points and the effective sample size is <30.

** CI of adjusted PR does not include 1.

**TABLE 2:** Prevalence of meeting the transition planning measure and individual indicators among adolescents aged 12–17 years, by mental, behavioral, or developmental disorder (mental, behavioral, or developmental disorder [MBDD] category and individual condition) status — National Survey of Children’s Health, United States, 2016–2017

Characteristic (no.)	Composite measure*		Time alone with PCP		PCP worked with adolescent		PCP discussed shift	
	%(95% CI)	aPR^†^ (95% CI)	% (95% CI)	aPR (95% CI)	% (95% CI)	aPR (95% CI)	% (95% CI)	aPR (95% CI)
**No MBDD (21,664)**	14.2 (13.2–15.3)	Ref.	36.8 (35.3–38.3)	Ref.	59.7 (58.0–61.4)	Ref.	50.8 (49.2–52.5)	Ref.
**Any MBDD^§^ (7,622)**	15.8 (14.1–17.5)	1.12 (0.99–1.27)	43.3 (40.7–46.0)	1.18^¶^ (1.10–1.27)	64.2 (61.4–66.9)	1.08 (1.02–1.13)	45.7 (43.0–48.4)	0.90 (0.85–0.96)
MBDD category**								
**Behavioral disorder (4,354)**	14.7 (12.5–17.1)	1.08 (0.92–1.28)	41.8 (38.5–45.1)	1.16 (1.07–1.26)	63.9 (60.4–67.3)	1.07 (1.01–1.14)	43.9 (40.4–47.4)	0.88 (0.81–0.96)
ADHD (3,612)	14.5 (12.1–17.2)	1.07 (0.89–1.29)	43.2 (39.5–46.9)	1.20 (1.10–1.31)	65.1 (61.1–68.9)	1.10 (1.03–1.17)	41.3 (37.4–45.3)	0.84 (0.76–0.92)
Behavior/Conduct problem (2,083)	13.5 (10.8–16.7)	1.03 (0.82–1.29)	37.9 (33.4–42.7)	1.07 (0.95–1.21)	58.4 (52.9–63.7)	0.99 (0.90–1.08)	45.7 (40.5–50.9)	0.93 (0.83–1.04)
Tourette syndrome (107)	12.4^††^ (4.7–25.0)	0.82 (0.37–1.84)	48.9^§§^ (31.6–66.3)	1.29 (0.89–1.88)	74.1 (60.0–85.3)	1.24 (1.05–1.46)	48.8^††^ (31.4–66.4)	0.93 (0.66–1.33)
**Emotional disorder (4,117)**	20.4 (17.9–22.9)	1.36 (1.18–1.56)	47.8 (44.6–51.0)	1.26 (1.17–1.37)	68.0 (65.0–71.0)	1.13 (1.08–1.19)	46.9 (43.7–50.1)	0.90 (0.83–0.96)
Anxiety (3,651)	19.8 (17.2–22.5)	1.32 (1.14–1.53)	47.2 (43.8–50.6)	1.25 (1.15–1.36)	67.4 (64.1–70.6)	1.12 (1.06–1.19)	45.8 (42.4–49.1)	0.88 (0.81–0.95)
Depression (2,030)	26.8 (22.8–31.1)	1.69 (1.43–2.00)	54.1 (49.6–58.6)	1.39 (1.27–1.53)	71.8 (67.6–75.7)	1.19 (1.11–1.27)	51.1 (46.6–55.6)	0.95 (0.86–1.04)
**Developmental disorder (3,221)**	12.6 (10.6–14.9)	0.93 (0.78–1.11)	39.1 (34.9–43.5)	1.08 (0.96–1.22)	60.6 (56.2–64.9)	1.02 (0.94–1.10)	43.5 (39.3–47.7)	0.87 (0.80–0.96)
ASD (887)	8.9 (5.8–12.9)	0.65 (0.45–0.95)	39.5 (29.4–50.4)	1.09 (0.83–1.44)	55.8 (45.9–65.4)	0.94 (0.79–1.12)	40.1 (31.0–49.6)	0.81 (0.66–0.98)
Learning disability (2,499)	13.0 (10.7–15.7)	0.95 (0.78–1.16)	40.5 (35.5–45.6)	1.12 (0.98–1.27)	62.4 (57.4–67.1)	1.05 (0.97–1.14)	42.7 (38.0–47.5)	0.86 (0.77–0.96)
Intellectual disability (400)	10.1 (5.2–17.3)	0.69 (0.39–1.21)	24.0 (16.4–33.1)	0.64 (0.45–0.90)	55.8 (44.9–66.3)	0.93 (0.77–1.12)	50.3 (39.6–60.9)	0.98 (0.79–1.20)
Developmental delay (1,367)	10.1 (7.6–13.2)	0.75 (0.57–0.99)	28.8 (24.2–33.7)	0.80 (0.68–0.95)	55.8 (49.9–61.7)	0.94 (0.84–1.05)	43.7 (38.0–49.6)	0.88 (0.78–1.00)
Speech/Language disorder (863)	9.2 (6.1–13.2)	0.73 (0.51–1.04)	33.3 (22.8–45.1)	0.96 (0.69–1.33)	62.3 (53.9–70.2)	1.06 (0.93–1.20)	38.1 (30.1–46.7)	0.80 (0.66–0.97)

**Abbreviations:** ADHD = attention-deficit/hyperactivity disorder; aPR = age-adjusted prevalence ratio; ASD = autism spectrum disorder; CI = confidence interval; PCP = primary health care provider.

* The composite measure of transition planning comprises the three individual elements: Time alone with PCP, PCP worked with adolescent, and PCP discussed shift. If an adolescent met all three elements, they were considered to have met the transition planning measure.

^†^ Prevalence ratios adjusted for age (aPR); all comparisons using aPRs use the “No MBDD” group as the reference group.

^§^ Children with any current MBDDs were identified based on the question “Has a doctor or other health care provider ever told you that this child has (specified disorder)?”; if the parent responded affirmatively, a follow-up question asked whether the child currently had the specified disorder. The “Any MBDD” category included parent report of one or more of the following: anxiety problems, depression, attention-deficit/hyperactivity disorder (ADHD), behavioral or conduct problems, Tourette syndrome, autism spectrum disorder (ASD), learning disability, intellectual disability, developmental delay, and speech or other language disorder. The clinical diagnosis of developmental delay (global developmental delay) is reserved for persons aged <5 years and requires reassessment for another diagnostic determination after a given period of time. Parent report of developmental delay in response to NSCH survey questions does not reflect a clinical diagnosis of developmental delay in adolescence.

^¶^ CI does not include 1.

** Individual MBDDs and MBDD categories are not mutually exclusive.

^††^ Estimate does not meet National Center for Health Statistics standards of precision and should be interpreted with caution. This percentage has a relative CI width >130%.

^§§^ Estimate does not meet National Center for Health Statistics standards of precision and should be interpreted with caution. The absolute width of the 95% CI is >30 percentage points.

The highest percentage of adolescents meeting the transition planning measure was those with emotional disorders (20.4%), specifically depression (26.8%). The lowest percentage of adolescents was those with developmental disorders (12.6%), specifically ASD (8.9%). Among adolescents with MBDDs, neither the presence of two or more co-occurring MBDDs (aPR = 1.07; 95% CI = 0.86–1.33) nor MBDD severity (aPR = 1.01; 95% CI = 0.82–1.25) was associated with meeting the transition planning measure. Adolescents with MBDDs who received treatment were more likely to meet the transition measure than were those with MBDDs who did not receive treatment (aPR = 1.38; 95% CI = 1.09–1.74) (Table 3).

**TABLE 3:** Adolescents with mental, behavioral, and developmental disorders (MBDDs) aged 12–17 years, meeting the composite transition planning measure and individual indicators, by MBDD co-occurrence, treatment status, and severity (unweighted n = 7,622) — National Survey of Children’s Health, United States, 2016–2017

Characteristic (no.)	Composite measure		Time alone with PCP		PCP worked with adolescent		PCP discussed shift	
	% (95% CI)	aPR (95% CI)	% (95% CI)	aPR (95% CI)	% (95% CI)	aPR (95% CI)	% (95% CI)	aPR (95% CI)
**Co-occurrence of MBDDs**								
1 MBDD* (3,176)	15.0 (12.4–17.8)	Ref.	45.1 (41.1–49.2)	Ref.	62.0 (57.8–66.1)	Ref.	45.9 (41.8–50.1)	Ref.
≥2 MBDDs (4,437)	16.4 (14.2–18.7)	1.07 (0.86–1.33)	42.0 (38.4–45.6)	0.92 (0.81–1.04)	65.7 (62.0–69.2)	1.06 (0.97–1.15)	45.6 (42.1–49.3)	0.99 (0.88–1.11)
**Severity of MBDD^†^**								
Only mild MBDD (3,419)	15.4 (13.2–17.8)	Ref.	43.1 (39.4–47.0)	Ref.	64.4 (60.4–68.3)	Ref.	46.3 (42.4–50.2)	Ref.
≥1 moderate/severe MBDD (4,203)	16.1 (13.7–18.7)	1.01 (0.82–1.25)	43.5 (39.7–47.3)	0.99 (0.88–1.12)	64.1 (60.2–67.8)	0.99 (0.91–1.08)	45.2 (41.5–49.0)	0.96 (0.86–1.08)
**Treatment^§^ status among adolescents with MBDDs**								
No treatment (2,056)	12.6 (10.1–15.5)	Ref.	38.1 (33.4–42.9)	Ref.	55.9 (50.4–61.3)	Ref.	47.8 (42.7–52.9)	Ref.
Any treatment (5,548)	17.0 (15.0–19.3)	1.38^¶^ (1.09–1.74)	45.6 (42.4–48.8)	1.21 (1.05–1.39)	67.9 (64.8–70.9)	1.22 (1.09–1.35)	44.7 (41.5–48.0)	0.94 (0.84–1.07)

**Abbreviations:** aPR = age adjusted prevalence ratio; CI = confidence interval; PCP = primary health care provider.

* Children with current MBDDs were identified based on the answer to the question “Has a doctor or other health care provider ever told you that this child has (specified disorder)?”; if the parent responded affirmatively, a follow-up question asked whether the child currently had the specified disorder. Any disorder included parent report of one of the following: anxiety problems, depression, attention-deficit/hyperactivity disorder (ADHD), behavioral or conduct problems, Tourette syndrome, autism spectrum disorder (ASD), learning disability, intellectual disability, developmental delay, and speech or other language disorder.

^†^ For each current MBDD indicated, the parent reported whether the MBDD was “mild,” “moderate,” or “severe.”

^§^ Treatment was indicated if the parent reported that the child with an MBDD was currently receiving medication treatment (i.e., medication for ADHD or ASD, or had taken any medication for emotional, concentration, or behavioral difficulties in the past 12 months) or behavioral services (i.e., speech, occupational, or behavioral therapy, treatment or counseling from a mental health professional in the past 12 months, or behavioral treatment for ADHD or ASD in the past 12 months).

^¶^ CI does not include 1.

## Discussion

Consistent with recent findings (*2*,*3*), this study found that a minority of U.S. adolescents receive recommended transition planning. Overall, rates of transition planning are higher among adolescents aged 15–17 years than among their younger peers (aged 12–14 years) suggesting that PCPs might be addressing transition to adult care as the transition becomes imminent. However, among adolescents aged 15–17 years, only 21.5% of those without MBDDs and 19.5% of those with MBDDs were receiving transition planning guidance, indicating a significant gap in transition planning for all adolescents.

Three subgroups of adolescents with MBDDs might be especially vulnerable to transition planning gaps. First, adolescents with ASD and other developmental disorders were least likely to meet the transition measure, suggesting that PCPs should work with families to better address the transition needs of these adolescents (*3*). Second, although adolescents with behavioral and emotional disorders had similar or higher levels of transition planning than did adolescents without MBDDs, only one in five adolescents with emotional disorders, and one in seven adolescents with behavioral disorders met the transition measure. Adolescents with behavioral and emotional disorders are at increased risk for disengagement from health care services during the transition to adult care, which can result in poor health outcomes (*1*,*6*,*8*). Finally, fewer than 20% of adolescents receiving medication, behavioral treatment, or both for MBDDs met the transition planning measure; this group might benefit from an increased emphasis on transition planning. Treatment continuity from adolescence into young adulthood is critical to long-term mental and physical health, and support during transition can increase the likelihood of maintaining adherence to current treatment (*1*,*4*,*5*). Together, these findings suggest increased attention to the transition needs of adolescents with MBDDs is warranted.

The findings in this report are subject to at least five limitations. First, the data are cross-sectional, so causality cannot be ascertained. Second, NSCH response rates were relatively low and might lead to nonresponse bias; however, sampling weights were applied to address nonresponse and produce nationally representative estimates. Third, indicators rely on parent-reported data, which might be subject to recall or social desirability bias; in addition, parents might not be aware of information provided during private discussions between adolescents and PCPs. Fourth, some PCPs may delay transition planning for adolescents in their care. Finally, treatment indicators might not record all treatments received.

All adolescents, especially those with MBDDs, could benefit from receiving earlier transition planning as recommended (*1*). Those with MBDDs might also benefit from condition-specific transition protocols with extended transition timelines, modified transition goals, and increased opportunities for comanagement between pediatric and adult PCPs (*1*,*9*). School-based transition programs and treatment appointments, including medication checks, provide opportunities outside preventive visits for transition planning work (*10*). Improving access to comprehensive and coordinated programs and services, as well as increasing provider training concerning adolescents’ unique mental and physical health care needs (*7*), could help increase the number of adolescents benefiting from successful health care transitions (*4*).

**Summary**

**What is already known about this topic?**

Adolescents with diagnosed mental, behavioral, and developmental disorders (MBDDs) are likely to disengage from and experience gaps in health care as they approach adulthood.

**What is added by this report?**

During 2016–2017, approximately 15% of adolescents, regardless of MBDD status, received transition guidance from their health care provider, but only 10% of adolescents aged 12–14 years received guidance. Among all adolescents with MBDDs, approximately 20% of those with emotional disorders and 13% of those with developmental disorders met the transition planning measure.

**What are the implications for public health practice?**

Improving access to comprehensive and coordinated programs and services, as well as increasing provider training concerning adolescents’ unique mental and physical health care needs, could help increase the number of adolescents benefiting from successful health care transitions.

## Footnotes

* The clinical diagnosis of developmental delay (global developmental delay) is reserved for persons aged <5 years and requires reassessment for another diagnostic determination after a given period. Parent report of developmental delay in response to NSCH survey questions does not reflect a clinical diagnosis of developmental delay in adolescence.

† For example, HRSA MCHB Adolescent and Young Adult programs: https://mchb.hrsa.gov/maternal-child-health-topics/adolescent-and-young-adult-health.

§ https://mchb.hrsa.gov/data/national-surveys/data-user.

¶ These categories are not mutually exclusive.

** Four survey items were used to measure the three transition elements: 1) At his or her last preventive check-up, did this child have a chance to speak with a doctor or other health care provider privately, without you or another adult in the room? (Time alone with HCP); 2) Has this child’s doctor or other HCP actively worked with this child to a) gain skills to manage his or her health and health care? (e.g., by understanding current health needs, knowing what to do in a medical emergency, or taking medications he or she may need?) or b) understand the changes in health care that happen at age 18 years (e.g., by understanding changes in privacy, consent, access to information, or decision-making) (HCP worked with adolescent); 3) [Has this child’s doctor or other HCP] talked with you about having this child eventually see doctors or other HCPs who treat adults? (HCP discussed shift).

†† https://mchb.tvisdata.hrsa.gov/PrioritiesAndMeasures/NationalPerformanceMeasures.

§§ The survey item on which this element is based was asked only for adolescents with a preventive visit in the past 12 months. Adolescents with no preventive visit were coded for analyses as not meeting this element.

¶¶ A total of 71,811 adolescents were included in the NSCH in 2016 and 2017. Additional information about response rates for the 2016 and 2017 NSCH can be found at https://www.census.gov/content/dam/Census/programs-surveys/nsch/tech-documentation/methodology/NSCH-2016-FAQs.pdf (2016) and https://www.census.gov/content/dam/Census/programs-surveys/nsch/tech-documentation/methodology/2017-NSCH-FAQs.pdf (2017).

*** Adolescents with responses to at least seven of the 10 sets of MBDD questions and no reported MBDDs were included in the no MBDD group.

††† Both MBDD type and the transition planning measure were strongly associated with age. To address this association, age-adjusted prevalence ratios were calculated to provide more appropriate comparisons across groups.
