# *Erratum:* Vol. 69, No. 34

**DOI:** 10.15585/mmwr.mm6943a6

**Published:** 2020-10-30

In the report “Support for Transition from Adolescent to Adult Health Care Among Adolescents With and Without Mental, Behavioral, and Developmental Disorders — United States, 2016–2017,” on page 1159, the third sentence in the Discussion should have read “However, among adolescents aged 15–17 years, only 21.5% of those **with** MBDDs and 19.5% of those **without** MBDDs were receiving transition planning guidance, indicating a significant gap in transition planning for all adolescents.”

